# Efferocytosis and prostate cancer skeletal metastasis: implications for intervention

**DOI:** 10.18632/oncoscience.440

**Published:** 2018-06-29

**Authors:** Hernan Roca, Laurie K. McCauley

**Affiliations:** Department of Periodontics and Oral Medicine, University of Michigan, School of Dentistry, Ann Arbor, MI 48109-1078, USA

**Keywords:** prostate cancer, skeletal metastasis, macrophage, efferocytosis, inflammation

**Figure 1:**
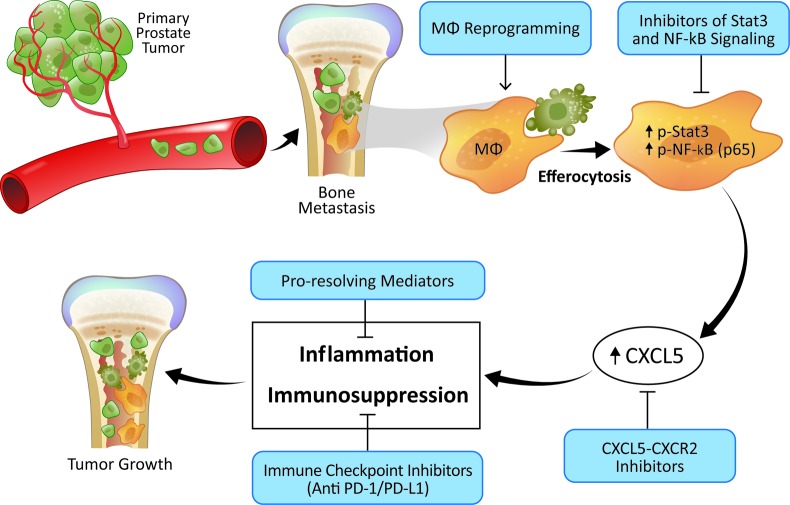
Potential intervention strategies (shown in blue) to target the cell death-accelerated tumor growth in bone metastatic prostate cancer

When tumor cells disseminate to the skeleton, they are bathed in a rich milieu of hematopoietic cells. Bone marrow myeloid cells as resident macrophages appear poised to engulf apoptotic tumor cells in a manner similar to when they engulf normal apoptotic cells during development and homeostasis, a process termed efferocytosis. Intriguingly we identified a different program of events in a macrophage when it engulfs an apoptotic cancer cell versus a non-cancer cell [1]. Upon efferocytosis of an apoptotic cancer cell NF-κB and Stat3 transcriptional machinery was activated and led to pro-inflammatory cytokine production, especially CXCL5, versus the anti-inflammatory cytokines normally attributed to efferocytosis. The resulting pro-inflammatory environment fueled further cancer cell growth hence implicating apoptotic cell clearance via tumor-associated macrophages in supporting tumorigenesis. Such a destructive cascade has previously been hinted at [2] but the entirety of the events and the human data to support it was described for the first time for prostate cancer skeletal metastasis [1]. Importantly, this destructive cascade provides clues for potential therapeutic intervention as suggested in (Figure 1).

An attractive potential therapy is based on the hypothesis that a macrophage can be reprogrammed towards an anti-tumor phenotype *in vivo*. Such an example was reported via inactivation of phosphatidylinositol 3-kinase-γ (PI3Kγ), which led to activation of cytotoxic CD8+ T-cells and tumor regression [3]. PI3Kγ signaling in macrophages acts as a molecular rheostat to inhibit NF-κB activation and inflammation in different mouse carcinoma models, while promoting immune suppression. It could be of interest to determine the impact of efferocytosis on PI3Kγ expression and whether inhibition of PI3kγ could suppress the cell death-accelerated growth of bone metastatic prostate cancer *in vivo*. A recent study shows how hematopoietic cell kinase (Hck; a Src family kinase) accelerates the growth of colon cancer by inducing the tumor-promoting M2-like macrophage polarization [4], further illustrating the potential of macrophage reprogramming. Pharmacological inhibition of HcK suppressed M2-macrophage polarization and tumor growth. This kinase, mainly expressed in B-lymphocyte and myeloid phagocytic lineages, regulates phagocytosis, which renders it of interest as a target in the efferocytic support of metastatic prostate cancer.

In addition to NF-kB activation, the Stat3 activation state of macrophages deserves special attention since simultaneous NF-κB and Stat3 activation correlates with tumor promoting activity. Given the importance of Stat3 signaling in the pro-tumoral function of macrophages it is tempting to speculate that inhibition of PI3Kγ would induce tumor regression in correspondence with low Stat3 activity, and even more effective anti-tumor responses could be achieved with both PI3kγ and Stat3 inactivation. Both Stat3 and NF-κB are persistently activated in cancer-associated immune cells and are intimately related to resistance to targeted therapies. Direct inactivation of Stat3 signaling has been achieved in mice via *in vivo* delivery of siRNA that targets Stat3 linked to CpG oligonucleotide agonist of toll-like receptor 9 (TLR9) [5]. This approach proved to be effective in silencing Stat3 in macrophages and induced anti-tumor immune responses. Current efforts are underway to identify the macrophage receptor(s) that are critical inducers of efferocytosis signaling mechanisms. One candidate is milk fat globule-EGF factor 8 (MFGE8) a bridging factor associated with Stat3 activation upon efferocytosis and is found in high levels in exosomes from prostate cancer patients [6]. The elucidation of other critical receptors will open new possibilities to dampen Stat3 and NF-kB.

Another intervention strategy would be to inhibit CXCL5-CXCR2 signaling, since CXCL5 increased with macrophage efferocytosis of apoptotic tumor cells and is increased in patients with prostate cancer metastasis [1]. Pharmacologic inhibitors of the CXCL5 receptor, CXCR2, have been used in clinical trials to prevent transplant rejection, to treat asthma and chronic obstructive pulmonary disease and their use may be extrapolated to bone metastasis. Although it is possible that inhibiting only the CXCL5-CXCR2 axis would be insufficient to be effective alone, there are studies of therapeutic intervention of a single axis, for example the CCL5-CCR5 in colorectal cancer metastases, that resulted in significant benefits inducing macrophage reprogramming and macrophage-mediated tumor regression [7].

Since efferocytosis of prostate cancer cells induces the activation of an inflammatory profile in macrophages, the use of pro-resolving mediators including lipoxins, resolvins and others could represent important points of intervention in metastatic prostate cancer. These mediators are normally released by immune cells to resolve inflammation and restore tissue homeostasis, and hence could accelerate the clearance of dying cancer cells while activating anti-inflammatory signaling to decelerate tumor progression. A recent work showed that resolvins inhibited tumor growth via enhancing the clearance of dead cells [8], supporting the use of pro-resolving mediators as a potential intervention strategy. The effectiveness of these treatment strategies in the context of bone and other metastases remains to be investigated.

Since immunosuppression is a common denominator of tumor growth in different cancer types, novel therapies that reactivate the patient's immune system are emerging as potent strategies in the battle against cancer. Success in the clinical use of immune checkpoint inhibitors in different tumor types are great examples of their potential, and additional combinatorial therapies will enhance the effectiveness of these inhibitors. Our results present new insights to the mechanisms that provide tumor cell advantages in the skeletal microenvironment and open new possibilities to strategically deplete tumor cells while re-programming macrophages to clear away dead cells leaving an environment that allows for resolution.
